# WRN helicase structural flexibility showcased through fragment-based lead discovery of inhibitors

**DOI:** 10.1063/4.0000901

**Published:** 2025-10-27

**Authors:** Rachel L Palte, Mihir Mandal, Justyna Sikorska, Artjohn Villafania, Meredith M Rickard, Robert Bauer, Xiaomei Chai, Jiafang He, Zahid Hussain, Markus Koglin, Hannah B MacDonald, My S Mansueto, Klaus Maskos, Joey L Methot, Aileen Soriano, Marcel J Tauchert, Sriram Tyagarajan, Minjia Zhang, Daniel J Klein, Jacqueline D Hicks, David G McLaren, Sandra B Gabelli, Daniel F Wyss

**Affiliations:** 1 Merck & Co., Inc., Boston, MA, USA; 2 Merck & Co., Inc., Rahway, NJ, USA; 3 MSD, London, England, United Kingdom; 4 Proteros Biostructures, Planegg-Martinsied, Germany, Germany; 5 Merck & Co., Inc., West Point, PA, USA

## Abstract

WRN helicase has been recognized as a promising synthetic lethal target for therapeutic intervention in cancers characterized by microsatellite instability-high (MSI-H) and mismatch repair deficiency (MMRd). However, the search for effective helicase inhibitors poses significant challenges, as high-throughput biochemical screening often yields limited validated candidates, many of which show poor activity in cellular contexts. In this study we show the power of non-covalent fragment-based lead discovery in locating new druggable allosteric sites on WRN and enabling us to combat the challenging behavior of WRN during high-throughput screening hampering hit identification. Through the fragment optimization process the structural enablement of WRN with key prioritized fragments revealed multiple conformations of WRN with significant domain rotations up to 180°, including a previously uncharacterized conformation. Rooted in a combination of biochemical, biophysical, and structural approaches, we present the detailed analyses of optimized chemical matter evolved from screening hits and the unique structural abilities of WRN to accommodate diverse conformations.

